# Deep Neural Networks Based on Span Association Prediction for Emotion-Cause Pair Extraction

**DOI:** 10.3390/s22103637

**Published:** 2022-05-10

**Authors:** Weichun Huang, Yixue Yang, Zhiying Peng, Liyan Xiong, Xiaohui Huang

**Affiliations:** School of Software Department, East China Jiaotong University, Nanchang 330013, China; hwc@ecjtu.edu.cn (W.H.); 2020068081200010@ecjtu.edu.cn (Z.P.); xly_ecjtu@163.com (L.X.); 2854@ecjtu.edu.cn (X.H.)

**Keywords:** emotion-cause pair extraction, multi-task learning, deep neural network

## Abstract

The emotion-cause pair extraction task is a fine-grained task in text sentiment analysis, which aims to extract all emotions and their underlying causes in a document. Recent studies have addressed the emotion-cause pair extraction task in a step-by-step manner, i.e., the two subtasks of emotion extraction and cause extraction are completed first, followed by the pairing task of emotion-cause pairs. However, this fail to deal well with the potential relationship between the two subtasks and the extraction task of emotion-cause pairs. At the same time, the grammatical information contained in the document itself is ignored. To address the above issues, we propose a deep neural network based on span association prediction for the task of emotion-cause pair extraction, exploiting general grammatical conventions to span-encode sentences. We use the span association pairing method to obtain candidate emotion-cause pairs, and establish a multi-dimensional information interaction mechanism to screen candidate emotion-cause pairs. Experimental results on a quasi-baseline corpus show that our model can accurately extract potential emotion-cause pairs and outperform existing baselines.

## 1. Introduction

**Figure 1:**
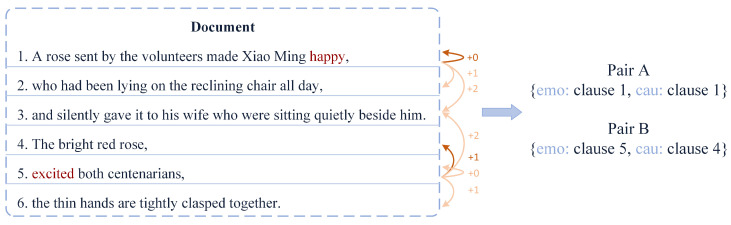
Document instances in the dataset.

“Emotion” has always been the focus of attention in the field of natural language processing. With the deepening of research, the potential causes behind emotions have received extensive attention from scholars. Emotion acquisition and its potential causes are widely used in e-commerce operations, public opinion orientation, and early warning of psychological abnormalities. To solve such problems, Lee et al. [1] proposed the emotion cause extraction (ECE) task. The ECE task aims to extract the underlying causes of emotion correspondence in a given document. However, this task needs to mark the emotion in the document in advance, which limits the application scope of the ECE task to a certain extent. In response to this problem, Xia and Ding et al. [2] proposed a new task, the emotion-cause pair extraction (ECPE) task, which aims to identify all potential emotions and their causes from unannotated documents. As shown in Figure 1, the document is divided into six clauses according to punctuation, where clause 1 and clause 5 contain the emotion words “happy” and “excited”, then clause 1 and clause 5 will be marked as emotion clauses. At the same time, the cause corresponding to “happy” is “A rose sent by the volunteers”, and the cause corresponding to “excited” is “The bright red rose”, so clause 1 and clause 4 will be marked as cause-clauses. Finally, through pairing learning, the output of the final task is the emotion-cause pair: Pair A {1,1}, Pair B {5,4}.

In order to improve the accuracy of emotion-cause prediction, Xia and Ding et al. [2] proposed a two-step framework (ECPE-2Step), which used multi-task learning to predict emotion clausesand cause-clauses, and pair them to form candidate emotion-cause pairs. Then, they used a filter to filter all possible emotion-cause pairs to obtain the final result. Experiments showed the effectiveness of the model, but the accuracy of the first step will directly affect the prediction results of the second step, thus reducing the prediction accuracy. In response to this situation, some scholars [3,4,5,6] proposed to use an end-to-end model to solve the ECPE task. Song et al. [7] used an end-to-end multi-task learning connection framework to solve this problem, regarding the ECPE task as a link prediction problem, and used the biaffine attention mechanism to judge whether there was a directional link between emotion and cause. Ding et al. [8] represented emotion-cause pairs in two dimensions and predicted them.

First, existing models fail to deal well with the potential relationship between the two subtasks and the emotion-cause pair task. Although the extraction of emotion clauses and cause-clauses is helpful for the pairing of emotion-cause pairs, it needs to be clear that, emotions and causes are the underlying information contained in the sentence itself. Therefore, we believe that mining the latent semantics of sentences in a targeted manner may be more conducive to the prediction of emotion-cause pairs. Learning the latent semantics of sentences can help to obtain information such as emotion and cause, as well as the connection between the two, thereby improving the prediction accuracy. At the same time, when mining and learning the semantic information of sentences, most scholars ignore the grammatical information contained in the text itself. As shown in Figure 1, the emotion “happy” in the document appears in sentence 1, and its corresponding cause “A rose sent by the volunteers” appears in the same sentence with a span of 0; the emotion “excited” appears in the sentence 5, and the cause “The bright red rose” appears in sentence 4, and the span is 1; both spans are less than or equal to 1. Therefore, according to this grammatical habit, we find that the causes generally appear within a certain range before and after the emotion.

Secondly, the complex pairing module will also cause data redundancy, with a large number of trainable parameters, resulting in excessive computational complexity, thereby reducing the prediction accuracy. Therefore, simplifying the model architecture and reducing model parameters is also one of the problems that current research needs to solve.

To address this issue, we propose a deep neural network model based on span association prediction for emotion-cause pair extraction, identifying effective emotion-cause pairs in an end-to-end manner. “Span association prediction” means using span information to enhance sentence semantic representation, information interaction, and prediction of emotion-cause pairs.The model utilizes a two-level mechanism to encode sentences in documents to obtain representation vectors for sentences. Then, we use a parallel mechanism to update the vector representations of all clauses using the span representation mechanism while making predictions for the emotion clauses and cause-clauses. The span representation mechanism can not only focus on the latent information in sentences, but also accurately capture the relationship between emotions and causes. Next, the obtained sentence representations are input into the span associated pairing mechanism for pairing. Finally, multi-dimensional information fusion is performed to obtain the final candidate pair representation vector, and then the final prediction is performed. Through the scope limit, irrelevant information is automatically filtered out from the model, making it clearer to obtain information. The span-based mode simplifies the model structure and reduces the amount of model parameters, thereby improving the operation accuracy.

The main contributions of this paper are as follows:

- We propose a span representation method for the ECPE task, which takes advantage of the idea of span association from the perspective of grammatical idioms;
- We designed a span-related pairing method to obtain candidate emotion-cause pairs, and establish a multi-dimensional information interaction mechanism to screen candidate emotion-cause pairs. At the same time, we simplified the model architecture and the number of trainable parameters was reduced;
- We experimented with our end-to-end model on a benchmark corpus, and the results showed that our method outperformed the state-of-the-art benchmarks.

The rest of this article is as follows. In the Section 2, the research progress of related scholars on the ECPE task is introduced in detail. The Section 3 mainly introduces the relevant details of the width correlation model. Section 4 presents the detailed experimental details and results analysis. Section 5 concludes the article and presents our outlook.

## 2. Related Work

### 2.1. Emotion Cause Extraction

Sentiment analysis is one of the key tasks in the field of natural language processing, and emotion cause extraction is a fine-grained task in the field of text sentiment analysis. Lee et al. [1] first utilized a word-level language rule system to detect causal events to solve the emotion cause extraction (ECE) task. Subsequently, many scholars [9,10,11,12,13] have used different ideas to study the ECE task. Russo et al. [14] proposed to extracting the underlying causes for emotion expression correspondence based on common sense. Yada et al. [15] proposed a bootstrapping technique to automatically acquire conjunctive phrases as textual cue patterns for emotion cause extraction. The proposed method first gathers emotion causes via manually given cue phrases. Hu et al. [16] adopted a way of pre-training a model based on an external emotion classification corpus for reinforcement emotion expression learning. Li et al. [17] adopted an attention-based RNN to capture the interconnectedness between emotion and causes clauses, and then, utilized a convolutional neural network to identify the underlying cause for emotion correspondence. Some work in other research areas has extracted cause in the context of multi-user microblogs [18,19,20,21]. Considering the important role commonsense knowledge plays in understanding implicitly expressed emotions and their causes, Turcan et al. [22] proposed a novel approach that combines commonsense knowledge with multi-task learning through an adaptive knowledge model to perform joint emotion classification and an emotion classification Reason tag. In addition, a model based on neural network architecture [23] was proposed to encode three elements (i.e., text content, relative position and global label) in a unified and end-to-end manner.

### 2.2. Emotion-Cause Pair Extraction

Emotion cause extraction (ECE) requires emotion labeling during prediction, which largely limits the application scenarios of ECE tasks. To address this issue, Xia et al. [2] first proposed the emotion-cause pair extraction (ECPE) task in 2019 and proposed a two-step framework to extract emotion clauses and cause-clauses, respectively, and input them into the model for training classification, thereby filtering out negative sentence pairs. However, this model also has certain drawbacks. To address these drawbacks, Wei et al. [3] proposed a model named RANKCP, which used a graph neural network to propagate between clauses to learn pairwise representations. The candidate emotion-cause pairs are sorted according to the learned pairwise representations, and finally predicted. Tang et al. [24] proposed a model named LAE-Joint-MANN-BERT, which is based on BERT for joint processing of emotion detection (ED) and ECPE tasks. Specifically, they calculated the attention value of the concatenated clauses in all clauses to indicate the relevance and importance of the concatenated clauses, thereby predicting the probability that each pair was an emotion-cause pair. Similarly, Fan et al. [25] transformed each given document into a directed graph and transform the original dataset into sequences, solving the ECPE task from different perspectives. Fan et al. [26] proposed an order-guided deep prediction model that integrated the different ordering between emotion clauses and cause-clauses into an end-to-end framework to tackle this task. Singh et al. [5] proposed an end-to-end model for the ECPE task, and adapted the NTCIR-13 ECE corpus, and established a baseline for the ECPE task on this dataset; the experiments demonstrated the effectiveness of their model. Chen et al. [27] took advantage of the rich interactions among the three tasks and performed multiple rounds of reasoning to repeatedly detect emotion, cause, and emotion-cause pairs, allowing the three tasks to effectively collaborate to improve prediction accuracy. In addition, through research on multi-task learning and deep learning [28,29,30,31], some scholars [32,33] have expanded the application scope of ECPE tasks and conducted experimental studies.

In the document processing method of ECPE task, many scholars use a two-level mode to process the input document, that is, they take the word as the initial embedding, and then use different motivation methods to process from word level to sentence level and sentence level to document level. This approach can handle the problem of two-level encoding well, while capturing the timing information contained in sentences and documents. Wei et al. [3] used a bidirectional RNN to perform secondary encoding on documents to obtain representations of sentences in documents. Singh et al. [5] utilized word-level Bi-LSTM and sentence-level Bi-LSTM to achieve two-level encoding of input documents.

## 3. Model

### 3.1. Problem Definition

We start with a document with multiple clauses, $D=\left\{S_{1},S_{2},S_{3},\cdots ,S_{n}\right\}$, where *n* is the number of sentences in the document. The emotion-cause pair extraction task aims to extract potential emotion-cause pairs from the given document:

(1) $$Pair=\left\{\cdots ,\left(S_{i}^{e},S_{i}^{c}\right),\cdots \right\},$$

where $\left(S_{i}^{e},S_{i}^{c}\right)$ represents the *i*-th emotion-cause pair. It should be noted that one emotion may correspond to multiple causes.

### 3.2. Overall Framework

**Figure 2:**
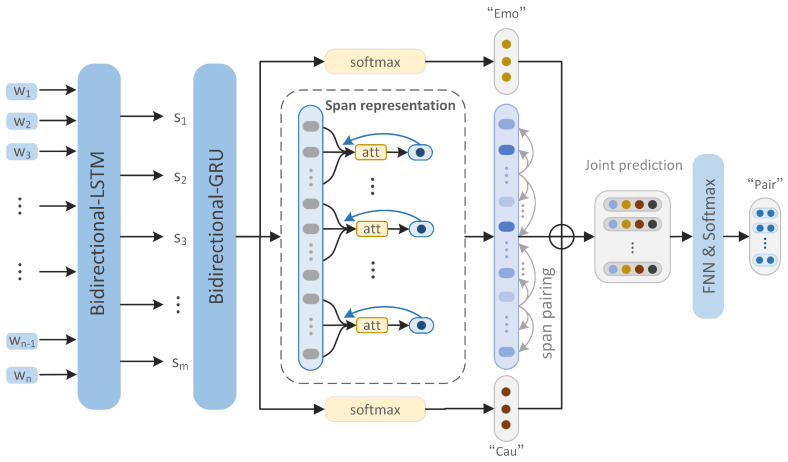
The general framework SAP-ECPE for ECPE tasks is introduced. The model consists of three parts, namely span representation, span pairing, and joint prediction, where Emo represents the prediction of the emotion clause and Cau represents the prediction of the cause-clause, span pairing represents the span association pairing module; and joint prediction represents the multidimensional information joint prediction module.

We propose a novel framework aimed at identifying different types of emotion-cause pairs (one-to-one and one-to-many) using deep neural networks based on span association prediction, and to reduce prediction errors while improving model efficiency. Our method mainly consists of three parts. First is the span representation part. We use Bi-LSTM and Bi-GRU to perform two-level encoding of sentences in documents, from words to sentences, and then from sentences to documents. The obtained sentence vectors are listed one by one, and each clause is used as a pivot to pay attention to its context information in many aspects. Then, the clauses are enhanced with targeted information to strengthen the causal relationship between sentences. Second is the span association pairing part. Through the obtained sentence vector, we pair the sentences in the document one by one in different spans with each clause as the pivot, and integrate the span information into the vector representation. Finally is the emotion-cause pair prediction part. We integrate the prediction results of emotion and cause, as well as the relative position and other information, and then use the multi-dimensional information interaction mechanism to screen and predict emotion-cause pairs. The details are shown in the Figure 2. Next, the main parts of the model will be described in detail.

### 3.3. Span Representation

**Figure 3:**
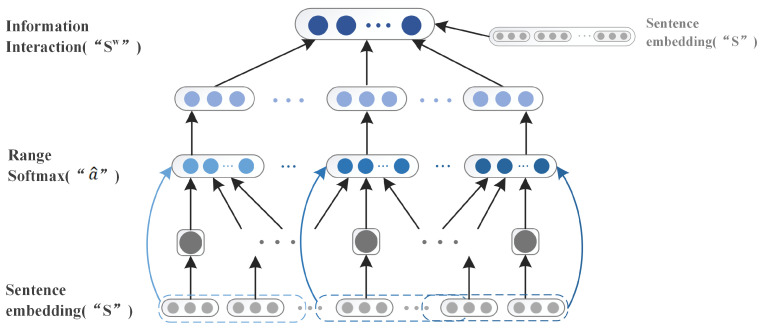
The processing based on span representation is described in detail, where *S* represents the sentence representation vector output by Bi-GRU.

In previous work, the Span-based model was applied in named body recognition, cleverly solving the problems of coverage and discontinuity in Named Entity Recognition (NER) [34]. We believe that the coverage and discontinuity problem in NRE is similar to the emotion and cause discontinuity problem of the ECPE task; therefore, we consider applying the span-based model to the emotion-cause pair extraction task to better address the emotion and cause pairing problems, that is, the one-to-one and one-to-many problems. Span representation combines context-dependent boundary representation with head-spotting attention mechanisms over spans [35]. We apply the span representation to the information learning of emotion and cause as an update mechanism to enhance the information interaction between sentences, as shown in Figure 3. Specifically, given a document $D=\left\{S_{1},S_{2},S_{3},\cdots ,S_{n}\right\}$, the sentence vector $S_{n}$ updated by Bi-GRU is operated as follows to update the sentence information:

(2) $$a_{n}=FNN_{s}^{*}\left(S_{n}\right),$$

(3) $${\hat{a}}_{i,n}=\frac{exp\left(a_{n}\right)}{\sum_{t=Span^{start}\left(i\right)}^{Span^{end}\left(i\right)}exp\left(a_{t}\right)},$$

(4) $$S_{n}^{w}=\sum_{t=Span^{start}\left(i\right)}^{Span^{end}\left(i\right)}{\hat{a}}_{i,n}⨀S_{n},$$

where $FNN_{s}^{*}$ represents a three-layer feedforward neural network, ${\hat{a}}_{i,n}$ represents the attention weight of each clause within a certain range, and $S_{n}^{w}$ represents the vector representation of the clause after the information interaction.

The obtained sentence representation is fused with the sentence representation vector $S_{n}$ output by Bi-GRU to obtain the span *w*, which is described in detail in Section 4.4. Finally, we obtain the updated representation vector $X_{n}$ of the sentence:

(5) $$X_{n}=add[S_{n},S_{n}^{w}].$$

### 3.4. Span Association Pairing

The model needs to extract effective emotion-cause pairs. First, it needs to solve two problems. One is semantic learning. We use the span representation method to solve this problem. The second is how to pair sentences in a document in the most efficient way. According to general language habits, the cause corresponding to an emotion is generally within a certain range before and after the emotion clause, and even appears in the same sentence as the emotion. Therefore, with this intuition, we take each sentence in the document as a hub, pair it with surrounding sentences within a certain span, and fuse information such as the relative positions to obtain the vector representation of the final candidate pair.

(6) $$E_{i}=FNN_{i}\left(W^{e}S_{i}^{emo}+b^{e}\right),$$

(7) $$C_{i}=FNN_{i}\left(W^{c}S_{i}^{cau}+b^{c}\right),$$

where $W^{e}$ and $W^{c}$ are the weight matrices, respectively, and $b^{e}$ and $b^{c}$ are the bias terms, respectively.

Through the span representation, the update vector representation *X* of the sentence is obtained, and the clauses in the span *w* are paired. The span $\left(i,j\right)$ indicates that the clauses at position *i* and position *j* are paired. Therefore, emotion-cause pairs can be expressed as:

(8) $$Pair_{i,j}=[X_{i},X_{j}],i\in [1,n],j\in [i-w,i+w+1].$$

Finally, the obtained information needs to be fused to provide multi-faceted information support for the prediction of emotion-cause pairs. Therefore, we fuse emotion and cause predictions with candidate emotion-cause pair representations, while adding relative position information to improve prediction accuracy. The specific formula is as follows:

(9) $$P_{i,j}=[Pair_{i,j},E_{i},C_{i},loc_{i,j}].$$

In the pairing process (Algorithm 1), since, according to the grammatical information, the causes generally appear within a certain range before and after the emotion, so our model uses each emotion clause as the pivot to perform cyclic pairing within a certain span (Lines 1–4). At the same time, in order to strengthen the degree of association between sentences and provide multi-dimensional information to the model, we add the prediction results of emotion and cause and the relative position information based on the span. (Lines 5–7).

**Algorithm 1** Span association pairing algorithm.**Input:** An input sentence $X=\left\{X_{1},X_{2},X_{3},\cdots ,X_{n}\right\}$**Output:** The candidate pair *P*1: **for** *i* **in** MAXDOCUMENTLENGTH **do**2:   **for** *j* **in** [i−w,i+w+1] **do**3:      **if** *j* **in** MAXDOCUMENTLENGTH **then**4:         $Pair_{i,j}\leftarrow \langle X_{i},X_{j}\rangle$5:      $loc_{i,j}\leftarrow U_{i,j}$6:      $Y_{i,j}^{pair}\leftarrow \langle E_{i},C_{i}\rangle$7:$P\leftarrow \left(Pair\cup Y^{pair}\cup loc\right)$8:**Return** *P*

### 3.5. Emotion-Cause Pair Prediction

Through span association pairing, we obtain the vector representation P of all candidate emotion-cause pairs $s_{i}^{emo}-s_{i}^{cau}$; then we use a feedforward neural network to combine softmax for prediction:

(10) $${\hat{P}}_{i,j}=FNN_{pair}\left(W^{pair}P+b^{pair}\right),$$

During the training process, we employ multi-task learning to jointly train the model. We use the cross-entropy loss function to calculate each task, that is, the emotion extraction task, the cause extraction task and the emotion-cause pair extraction task:

(11) $$L^{emo}=-\sum_{i=1}^{\left|n\right|}y_{i}^{emo}\cdot log\left(E_{i}\right),$$

(12) $$L^{cau}=-\sum_{i=1}^{\left|n\right|}y_{i}^{cau}\cdot log\left(C_{i}\right),$$

(13) $$L^{pair}=-\sum_{i=1}^{\left|n\right|}\sum_{j=1}^{\left|s\right|}y_{i,j}^{pair}\cdot log\left({\hat{P}}_{i,j}\right),$$

where $y_{i}^{emo}$, $y_{i}^{cau}$, and $y_{i,j}^{pair}$ are the true values of emotion, cause, and emotion-cause pair, respectively.

We sum the three to get the loss function of the final model:

(14) $$L=L^{pair}+L^{emo}+L^{cau}+\lambda {∥\theta ∥}^{2},$$

where λ represents the weight of the $l_{2}$ regularization term, and θ is the $l_{2}$ regularization term of all parameters in the model. For more training details, see the experiment.

## 4. Experiment

In this section, we evaluate the effectiveness and robustness of the model experimentally, and introduce more experimental details.

### 4.1. Implementation Details and Evaluation Metrics

We validate the effectiveness of the model using publicly available datasets for the ECPE task built by Xia and Ding. The dataset was annotated on the basis of the ECE task and has 1945 documents whose contents were from Sina City News. In the dataset, each sentence in the document was divided into several clauses, and the specific statistical information is shown in the Table 1. We took clauses as the model input to extract emotion-cause pairs at the sentence level. During the experiment, we divided the dataset into 10 folders, randomly selected nine folders as the training set and one folder as the test set, and repeated the experiment 10 times, and reported the average results.

We use Precision, Recall and the F1-score as metrics to measure our experimental results, calculated as follows:

(15) $$P=\frac{\sum correct\_ECPs}{\sum proposed\_ECPs},$$

(16) $$R=\frac{\sum correct\_ECPs}{\sum annotated\_ECPs},$$

(17) $$F1=\frac{2\times P\times R}{P+R}$$

where correct_ECPs represents the correct emotion-cause pair predicted by the model, proposed_ECPs represents the emotion-cause pair predicted by the model, and annotated_ECPs represents the emotion-cause pair marked in the dataset.

We used Word2vec [36] to pre-train on the Chinese Weibo corpus to obtain the corresponding word vectors. The dimensions of word embedding and relative position were set to 200 and 50, respectively. The number of hidden units of Bi-LSTM and Bi-GRU in the model were both set to 100. The span size in the span representation was set to seven. To prevent overfitting, dropout in the feedforward neural network was set to 0.5 and 0.9, respectively. L2-normalization was set to $1\times 10^{-5}$.

For training details, we used the stochastic gradient descent (SDG) along with Adam optimization with shuffled batches. The batch size and learning rate were set to 32 and 0.008, respectively. All weight matrices and biases were randomly initialized to a uniform distribution U(0.01, 0.01).

### 4.2. Baseline Models

We compared our model with the following baseline models:

- Indep: The first model proposed by Xia and Ding [2] is a two-step model. In the first step, emotion extraction and cause extraction are regarded as two independent tasks, respectively, and the emotion and cause are extracted through Bi-LSTM; in the second step, emotion and cause are paired and the classifier is used for binary classification.
- Inter-CE [2]: The general process of the model is the same as that of Indep. It is an interactive multi-task learning method that uses the prediction of cause extraction to strengthen emotion extraction.
- Inter-EC [2]: This is another interactive multi-task learning method that uses predictions from emotion extraction to reinforce cause extraction, the rest of the model is the same as Indep.
- E2EECPE: An end-to-end model proposed by Song et al. [7], this is a multi-task learning linking framework that exploits a biaffine attention to mine the relationship between any two clauses.
- ECPE-2D: Proposed by Ding et al. [8], tthis model realizes all the interactions of emotion-cause pairs in 2D, and uses the self-attention mechanism to calculate the attention matrix of emotion-cause pairs. Here, we choose the Inter-EC model with better effect.

### 4.3. Overall Performance

We calculated the performance analysis of all methods on the three tasks of emotion extraction, cause extraction, and emotion-cause pair extraction, respectively. As shown in the Table 2, among all the compared models, our proposed model achieves the best results in F1 of the three tasks. First, a comparative analysis was performed with the model of Xia and Ding [2] (Indep, Inter-EC and Inter-CE). Taking the relative best model Inter-EC among the three models as an example, our model outperformed its F1 score by 1.86%, 3.10% and 5.66% on the three subtasks, respectively. While Inter-EC takes full account of the correlation between emotion and cause, it increases the likelihood of error propagation as a staged model. The difference is that our model not only considers the correlation between emotion and cause, but also adjusts the ensemble with an end-to-end model, reducing error propagation.

Compared with the other joint models, our model outperformed the ECPE-2D model by 1.73% on the F1-score of the emotion-cause pair extraction task. We guess that ECPE-2D uses the Transformer to learn the relationship between pairs after pairing the predicted emotion and cause combinations, which ignores the direct information contained in the sentence itself. In contrast, our model strengthens the learning of sentence information, and strengthens sentence information with emotion as the pivot according to general language habits, thereby achieving better information fusion and obtaining better experimental results. Not only that, our model also has 11.92% less parameter size than ECPE-2D. The details are shown in the Table 3. In addition, our model also achieved better results than the E2EECPE model, which may be caused by insufficient learning of emotion and cause.

### 4.4. Further Discussions

**Ablation experiment** To verify the effectiveness of the model, we conducted ablation experiments on each module of the model. The experimental results were compared one by one, and the results are shown in the Table 4.

First, we remove the span representation module. Compared with our model, we found that the score was significantly lower on the F1 metric, which can verified the effectiveness of the module. At the same time, it can be found that the accuracy P was improved in the model without the span representation module. Therefore, from another perspective, Span association pairing can improve the prediction accuracy of the model through precise positioning.

Second, we remove the span association pairing module. By analyzing the Table 4, it can be seen that the comprehensive index F1 has dropped significantly. However, the recall rate was significantly improved by 2.26%. We speculate that this may be the function of the span representation module, which strengthens the relationship between sentences and performs multi-scale fusion of sentence information, thereby improving the recall rate R of the model.

Along with the above analysis, our model combines the above two modules, starting from the precision and recall rate, respectively, the two modules interact, and improve the precision and recall rate at the same time, and finally improve the F1, which is better than the most Advanced Baseline Model.

**Figure 4:**
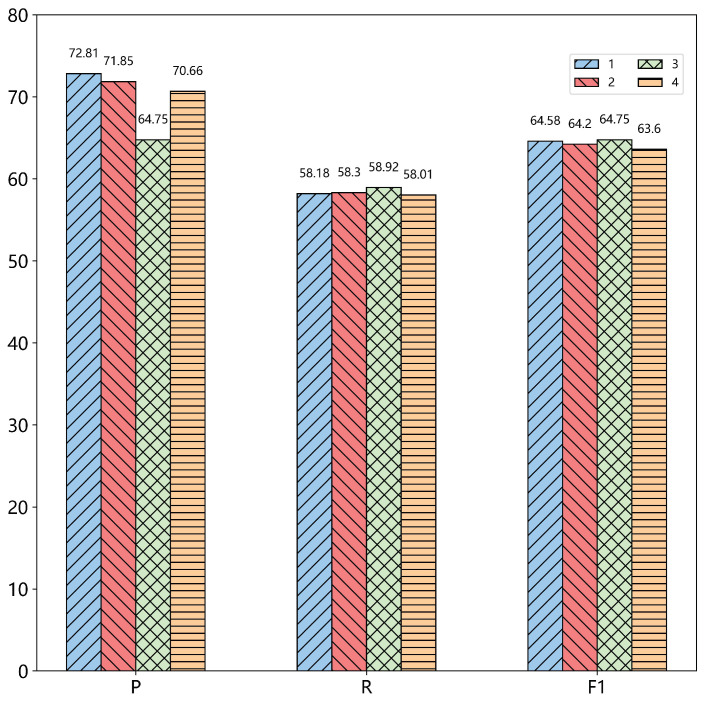
Precision, recall, and F1 value variation of ECPE tasks across different spans.

**Figure 5:**
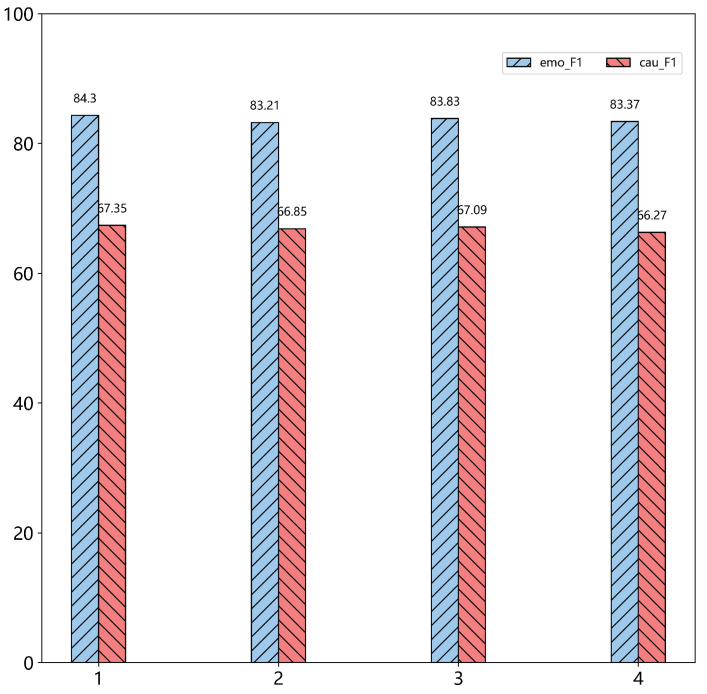
F1 changes of emotion clause extraction task and cause clause extraction task in different spans.

**Comparing with span selection** In the model, whether it is the module span representation or span association pairing, it is operated within a certain span. Thus, the selection of this span is particularly important, so we have conducted experiments with different widths, and the experimental results are shown in the Figure 4 and Figure 5.

It can be seen from the experiments that on the ECPE task, span 3 was significantly better than the other spans, so that P and R have a good balance, thus obtaining the optimal F1 value. We also analyzed two subtasks, and the performance on the two subtasks was not the same for different spans. Span 1 and Span 3 performed better in the two subtasks, but synthesizing the ECPE task, we finally choose Span 3 as the width of the model. According to the experimental results, we found that the cause for emotion correspondence was generally distributed in the three clauses before and after the clause where the emotion was located, and processing sentences within this range will obtain the best results.

## 5. Conclusions

The emotion-cause pair extraction (ECPE) task is a fine-grained sentiment analysis task that captures the emotion in documents and their underlying causes. In this paper, we proposed a deep neural network model based on span association prediction for emotion-cause pair extraction. Using the span representation method, from the perspective of grammatical habits, the idea of span was used to deal with this task. At the same time, a span association matching method was used to strengthen the study of the deep meaning of sentences, pair the sentences in the document, and accurately obtain the candidate emotion-cause pairs. At the same time, we simplified the model architecture and reduced the number of trainable parameters, thus improving the prediction accuracy of the model. In the future, we plan to conduct in-depth research on accurately capturing the latent information of sentences, and integrate methods such as a graph attention mechanism into our model to improve the prediction accuracy of the model. In future work, we hope to incorporate more advanced modules, such as a graph attention network, BERT, etc., to enhance semantic learning.

## Figures and Tables

**Table 1:** Statistics on the number of emotion-cause pairs in the dataset.

	Document	Percentage
ALL	1945	100%
1 pair	1746	89.77%
2 pairs	177	9.10%
≥3 pairs	22	1.13%

**Table 2:** Comparison of model results for ECPE task, emotion extraction and cause extraction. The maximum value is marked in bold.

	Emotion Ext			Cause Ext			Emotion-Cause Pair Ext			
Models	P(%)	R(%)	F1(%)	P(%)	R(%)	F1(%)	P(%)	R(%)	F1(%)	Δ
Indep	83.75	80.71	82.10	69.02	56.73	62.05	68.32	50.82	59.18	−7.02%
Inter-CE	84.94	81.22	83.00	68.09	56.34	61.51	69.02	51.35	59.01	−7.29%
Inter-EC	83.64	81.07	82.30	70.41	60.83	65.07	67.21	57.05	61.28	−3.72%
E2EECPE	85.95	79.15	82.38	70.62	60.30	65.03	64.78	**61.05**	62.80	−1.34%
ECPE-2D	84.63	**81.95**	83.19	**72.17**	62.66	67.01	71.31	57.86	63.65	0
**SAP-ECPE**	**86.31**	81.58	**83.83**	70.11	**64.42**	**67.09**	**72.18**	58.92	**64.75**	+1.73%

**Table 3:** Comparison of trainable parameters for SAP-ECPE and ECPE-2D.

Method	Trainable Parameters	Δ
SAP-ECPE	933,755	11.92%
ECPE-2D(Inter-EC)	1,060,116	0

**Table 4:** Results of ablation experiments on the model on the ECPE task. The maximum value is marked in bold.

Method	P(%)	R(%)	F1(%)	Δ
Ours w/o Span pepresentation	**72.43**	57.25	63.87	−1.36%
Ours w/o Span association pairing	67.37	**60.25**	63.54	−1.87%
Ours	72.18	58.92	**64.75**	0

## Data Availability

Not applicable.
